# Can Haematological and Hormonal Biomarkers Predict Fitness Parameters in Youth Soccer Players? A Pilot Study

**DOI:** 10.3390/ijerph17176294

**Published:** 2020-08-29

**Authors:** Fabrizio Perroni, Silvia Migliaccio, Paolo Borrione, Mario Vetrano, Stefano Amatori, Davide Sisti, Marco B. L. Rocchi, Gerardo Salerno, Riccardo Del Vescovo, Elena Cavarretta, Laura Guidetti, Carlo Baldari, Vincenzo Visco

**Affiliations:** 1 Department of Biomolecular Sciences, Section of Exercise and Health Sciences, University of Urbino Carlo Bo, 61029 Urbino, Italy; fabrizio.perroni@uniurb.it; 2 Department of Movement, Human and Health Sciences, University of Rome “Foro Italico”, 00135 Rome, Italy; silvia.migliaccio@uniroma4.it (S.M.); paolo.borrione@uniroma4.it (P.B.); laura.guidetti@uniroma4.it (L.G.); 3 Physical Medicine and Rehabilitation Unit, Sant’Andrea Hospital, “Sapienza” University of Rome, 00189 Rome, Italy; mario.vetrano@uniroma1.it; 4 Department of Biomolecular Sciences, Service of Biostatistics, University of Urbino Carlo Bo, 61029 Urbino, Italy; s.amatori1@campus.uniurb.it (S.A.); davide.sisti@uniurb.it (D.S.); marco.rocchi@uniurb.it (M.B.L.R.); 5 Department of Clinical and Molecular Medicine, Sant’Andrea Hospital, Faculty of Medicine and Psychology, “Sapienza” University of Rome, 00189 Rome, Italy; gerardo.salerno@uniroma1.it (G.S.); vincenzo.visco1@uniroma1.it (V.V.); 6 “Empoli F.C.” Soccer Team, 50053 Empoli, Italy; delvescovoriccardo@gmail.com; 7 Department of Medico-Surgical Sciences and Biotechnologies, Sapienza University of Rome, 04100 Latina, Italy; elena.cavarretta@uniroma1.it; 8 Mediterranea Cardiocentro, 80122 Napoli, Italy; 9 Faculty of Psychology, eCampus University, Novedrate, 22060 Como, Italy

**Keywords:** cortisol, countermovement jump, cytokines, maximal oxygen uptake

## Abstract

The study aimed to investigate the correlations among immune, haematological, endocrinological markers and fitness parameters, and assess if the physiological parameters could be a predictor of fitness values. Anthropometric, physical evaluations (countermovement jump—CMJ, 10 m sprint, VO_2_max, repeated sprint ability—RSA total time and index) and determination of blood (IL-6, IL-10, IL-17A and tumour necrosis factor) and salivary (testosterone and cortisol) samples parameters in 28 young male soccer players (age: 13.0 ± 0.2 years, body mass index (BMI): 19.5 ± 2.2 kg/m^2^) were analysed. To evaluate the dependence of the variables related to athletic performance, multiple linear regression with backward stepwise elimination was considered. A significant regression equation was found in CMJ (F_(5,16)_ = 9.86, *p* < 0.001, R^2^ adjusted = 0.679) and in the RSA index (F_(5,16)_ = 15.39, *p* < 0.001, R^2^ adjusted = 0.774) considering only five variables, in a 10 m sprint (F_(4,17)_ = 20.25, *p* < 0.001, R^2^ adjusted = 0.786) and in the RSA total time (F_(4,17)_ = 15.31, *p* < 0.001, R^2^ adjusted = 0.732) considering only four variables and in VO_2_max (F_(9,12)_ = 32.09, *p* < 0.001, R^2^ adjusted = 0.930) considering nine variables. Our study suggests the use of regression equations to predict the fitness values of youth soccer players by blood and saliva samples, during different phases of the season, short periods of match congestion or recovery from an injury.

## 1. Introduction

Several studies have reported that repeated physical and psychological stressors lead to haematological, inflammatory and endocrine changes, which result in adaptive physiological responses and affect the physical performance of sports players [1,2]. Furthermore, growing evidence supports the hypothesis that, during the young soccer player agonistic season, correct maintenance of homeostasis is finely regulated through the immune, haematological and endocrine systems interaction, and contributes to the athlete performance and physical integrity [3,4]. Therefore, several biochemical markers could be used as indicators for physical stress, systemic inflammation, muscle damage and physiological adaptation to sports activity [5,6,7].

In this regard, testosterone (T) and cortisol (C) play an important role in exercise-related stress. T is mainly involved in anabolic processes, which support healthy muscle development [8], while C is a strong indicator of catabolic metabolism [9]. The T/C ratio has been positively related to changes in physical performance and indicated as a balance of anabolic/catabolic activity [10], representing a useful tool in the early detection of overtraining [11]. Our group [12] found significant correlations between fitness and hormonal parameters in youth soccer players before the beginning of 8-weeks of the preseason training phase. Other authors [13,14] showed significant alterations in the hormonal concentrations in the young soccer players during the six-month of the competitive season. Despite its diffusion and popularity, very few studies have been carried out on evaluating biochemical, haematological and endocrine parameters metabolism of youth soccer players.

Concerning the exercise-induced inflammation, the role of some cytokines, proteins with several pleiotropic effects involved in cell signalling during inflammatory responses [15], should be better elucidated. Araujo et al. [16] suggested that physical training induces adaptations to the stress mediated by an immune response, resulting by changes in the cytokine profile. However, pro-inflammatory cytokines (including IL-6, TNF-alpha and IL-17AA) may be preferentially upregulated during acute exercise, while chronically prolonged physical activity may promote an anti-inflammatory status presumably associated with IL-10 secretion [3,4,14,17,18,19]. Furthermore, Heisterberg et al. [20] have shown that variations of haemoglobin and haematocrit were related to the amount of aerobic and anaerobic training, strength training and the number of matches per week in professional soccer players. Silva et al. [21] showed that soccer players face significant changes in biomarkers of physiologic strain (muscle damage and oxidative stress-related markers) during the competitive season, but values return to normal during the off-season.

The aim of the present study was to investigate if some biological parameters could be predictive of fitness values in youth soccer players, throughout the whole season. The majority of reports in this field were previously focused on measures of performance, progress in training and identifying overtraining [22]. To our knowledge, this is the first attempt to predict fitness values in order to provide trainers and sports physicians useful reports on performance and physiological adaptations of their soccer players in specific moments of a season (i.e., short periods of match congestion).

## 2. Materials and Methods

### 2.1. Participants

Twenty-eight young male soccer players (age 13.0 ± 0.2 years, height 166 ± 9 cm, weight 54.1 ± 11.0 kg, body mass index (BMI) 19.5 ± 2.2 kg/m^2^) recruited from a youth team of professional Soccer Club (A.S. Roma) volunteered to participate in this study. Inclusion criteria were: at least six years of competitive and training experience, training volume of at least four days (a minimum of four 1.5-h training sessions) and a 60-min match per week; exclusion criteria were: injuries or >10% of absence from training sessions (in the last month before the tests). Thus, 22 participants were included in the final analysis. Players were in good health and were not taking medication, nutritional supplements or drugs that could influence the experimental protocol, and they lived in their own homes with their respective families. Before the study, after verbal and written explanation of the experimental design of the study, a signed consent form was filled by parents of all soccer players (<18 years). In addition, they were informed that they could withdraw from the study at any time. The Bioethics Committee of the University of Turin (Study Protocol No. 134685) approved the study, and all procedures were in accordance with the ethical standards of the institutional and national research committee and with the 1964 Helsinki declaration and its later amendments.

Before the test session, all youth soccer players underwent the standardised training sessions developed by the technical club’ coaches during the preseason training phase. None of the participants underwent any strenuous activity and training outside of their regular training schedule. During the training season, a nutritionist planned each athlete’s diet.

### 2.2. Experimental Design

Measurements consisted of anthropometric and physical evaluations, and determination of blood and salivary sample parameters before the beginning of the Italian competitive soccer season (October). Each testing session was performed in three days with the same sequence (Day 1: blood and salivary sample collection and anthropometric measurements; Day 2: ‘Explosive Efforts’ and ‘High-Intensity Efforts’ evaluation and Day 3: aerobic evaluations). Blood sample collection and anthropometric assessments were organised in the morning (between 8:00 and 8:30 a.m.), while physical evaluations in the afternoon (between 4:00 and 5:00 p.m.). To minimise circadian rhythms and climate-related factors, training sessions and experimental evaluations were performed in similar environmental conditions (temperature: 18–20 °C; humidity: 50–60%) on an artificial turf, which is almost unaffected by weather conditions [23], approved for national-level competitions. These evaluations are considered by the staff of “A.S. Roma” Soccer Club as routine exams of their young soccer players, and they agreed to the participation of their players in the study. To reduce measurement variation, the same experienced investigator conducted all the evaluations. Subjects were instructed to avoid any relevant high-intensity activities 24 h before the testing session (they were free from training sessions) and to avoid food and drink in the hour before testing.

Once arrived at the training centre, each participant was conducted to the medical room where players were informed about the study aims and their anthropometric measurements (to enhance the positive engagement of participants), then blood and salivary samples were collected. Before starting the fitness tests, players underwent a standardised warm-up period consisting of jogging (40–60% of individuals’ theoretical maximal heart rate, calculated as 220-age and monitored by heart rate devices), strolling locomotion and dynamic stretching (15 min).

Considering the nature of the jumping and acceleration activities and the frequency with which they occur in a soccer match, we used the countermovement jump (CMJ) test [24], and the 10 m sprint test to evaluate the explosive capacity of the players. Numerous research has confirmed the validity and reliability of the 10 m sprint test using electronic timing gates [25,26]. The Yo-Yo Intermittent Recovery Test Level 1 (YYIRT1) [27] was used to estimate the Maximal Oxygen Uptake (VO_2_max) of each player. This test is considered a reliable and valid measurement of match-related fitness performance in soccer [28].

Given the amounts of intermittent sprinting and multidirectional changes of direction performed by soccer players [29,30,31] and the decrease of the amount of high-intensity activities toward the end of the match [30,32], we used the protocol by Bangsbo et al. [33] to evaluate the repeated sprint ability (RSA). 

CMJ, 10 m sprint and RSA tests were performed in temporal sequence. Although CMJ and 10 m sprint are explosive efforts with short duration, to avoid the effect of fatigue, we preferred to give an additional pause of 5 min between tests. During each test, soccer players were verbally encouraged to perform the tests with full concentration and maximum effort.

### 2.3. Anthropometric Evaluation

After shoes and heavy clothing were removed, weight (kg) and height (cm) were measured using an electronic scale and a stadiometer (Seca 702, Seca GmbH & Co. KG, Hamburg, Germany) with an accuracy of ± 0.1 kg and ± 0.1 cm, respectively. Body mass index (BMI) was calculated for each subject by dividing the weight by the square of the height (kg/m^2^).

### 2.4. Biochemical Collection and Evaluation

After that blood and saliva samples were taken, they were stored into a cooler bag and immediately transported to the laboratory (10 min of distance by car from training centre) and centrifuged (IEC FL 40, Thermo Scientific) at 4 °C, 3000 rpm for 10 min (blood sample) and 15 min (salivary sample).

Of the whole blood sample, 10 mL was drawn from a peripheral vein and collected in analytical tubes containing EDTA and, after centrifugation, plasma samples were then aliquoted and stored at −80 °C until analysis, so avoiding repeated thawing and freezing. Cytokine plasma levels (IL-6, IL-10, IL-17A and tumour necrosis factor (TNF-α)) were simultaneously measured by multiple immunoassay kits (Human Magnetic Luminex Assay, R&D System Inc. Bio-Techne brand, Minneapolis, MN, USA) following the manufacturer’s instructions and using the Luminex Technology. Quantitative data were obtained by the Luminex-200 MagPix system (Luminex Corporation, Austin, TX, USA), and the data were analysed using Luminex 200 software. Quantitative analysis of cytokine secretion was performed as mean fluorescence intensity (MFI) as above. Complete blood counts were determined by the CELL-DYN Sapphire Automated Hematology Analyzer (Abbott).

Players were asked to self-collect the saliva samples using a cotton swab and saliva collecting tube (DRG International Inc. USA) at the same time of the day (8:00–8:30 a.m.), in order to avoid the effects of the circadian rhythm and variations in food intake. During saliva collection, contamination with food debris was avoided by rinsing the mouth with water and by delaying the collection for 15 min after rinsing to prevent sample dilution. Following centrifugation, saliva samples were stored in the fridge at −20 °C until they were assayed. Saliva samples were analysed in duplicate to measure salivary testosterone (sT) and salivary cortisol (sC), using commercially available kits (DRG Diagnostics, Marburg, Germany). Each test was performed according to the manufacture protocol. The sensitivities of sT and sC assays were 2.63 pg/mL (range of detection between 0.94 and 1000 pg/mL) and 0.537 ng/mL (range of detection: 0.537–80 ng/mL), respectively. Inter- and intra-assay coefficients of variation were <8% and 5% for the measurements of sC, and <10% and 5% for the sT, respectively. 

### 2.5. Countermovement Jump (CMJ)

An optical acquisition system (Optojump, Microgate, Udine, Italy) was used to measure the explosive power of the lower extremities of soccer players through a CMJ test, according to the protocol described by Bosco et al. [24]. From the standing position, subjects had to quickly bend their knees to a 90° angle and, immediately after, to perform a maximal effort and explosive vertical jump. The hands were kept on the hips to avoid any effect of arm-swing, and trying to avoid any knee or trunk countermovement. During the flight phase, subjects had to keep their body vertical and land with feet together, and the knees almost fully extended [34]. The optical system was activated by the feet of the subject at the instant of taking-off (10^−3^ s of resolution); jump height was calculated in real-time by specific software [35]. Each subject performed three correct jumps with a 1-min passive pause in between, and the highest was taken for further analysis. If a participant failed to rigorously adhere to the protocol, the trial was repeated after an additional one-minute rest. 

### 2.6. Ten Meter Sprint

A dual infrared reflex photoelectric cells system (Polifemo, Microgate, Udine, Italy) was used to evaluate 10 m performances. The first timing gate was positioned at 0.5 m from the start. For each test, the subjects had to perform three trials with a 5-min recovery period between trials. The best performance was used for statistical analysis.

### 2.7. Aerobic Evaluation

VO_2_max of soccer players was estimated by the YYIRT1. [27]. The test required repeated 2 × 20 m shuttle runs between a start and finish line, at a progressively increased speed controlled by an audio metronome from a calibrated CD player. There was a 10 s period of active recovery (decelerating and walking back to the starting line) between runs. When a subject failed twice to reach the finishing line in time, the distance covered at that point was recorded and considered the test result [2]. VO_2_max was estimated by the formula [28]:

VO_2_max (mL/kg/min) = distance covered (m) × 0.0084 (mL/kg/min)/m + 36.4 (mL/kg/min) (1)

The YYIRT1 was performed in groups of 10–12 players.

### 2.8. RSA Test

The RSA test [33] included 7 × 30 m sprints, interspersed by 25 s of active recovery. Players were requested to perform the sprints to the best of their abilities, decelerating as quickly as possible after the finishing line. The recovery consisted of self-paced jogging to return to the starting line for a new start. A dual infrared reflex photoelectric cell system (Polifemo, Microgate, Udine, Italy) positioned 30 m from the start line was used to evaluate RSA performance. For the RSA test validation, the first sprint time had to be not slower than 5% of the individual’s best 30 m performance previously assessed [36,37,38]. If a participant failed to adhere to the protocol, it was repeated after an additional one-minute rest.

The sum of sprinting scores over 30 m was assumed as global RSA performance (total time, TT) [37,38], and a fatigue index (%) was calculated as follows [37]:

(fatigue index = ((total sprint time/lowest sprint time × 7) × 100) − 100).(2)

### 2.9. Statistical Analyses 

For quantitative variables, mean ± standard deviation, minimum–maximum values (min–max) and CV were reported. CV (standard deviation/mean, in percentage) is useful to compare the dispersion degree among variables, which have different units of measurement. To evaluate the dependence of the variables related to athletic performance (CMJ, 10 m sprint, VO_2_max, RSA total time and RSA index; dependent variables: DVs), multiple linear regression with backward stepwise elimination was considered. Predictors (independent variables: IVs) were: age (in months), BMI, haematic variables (IL-6, IL-10, IL-17A, TNF-α, PCR, LDH, CK, erythrocyte, leucocyte, haemoglobin, haematocrit and thrombocyte), salivary cortisol and testosterone levels. Stepwise regression does a multiple regression several times, each time removing the weakest correlated variable; at the end, are reported the variables that explain the distribution as best (*p* of F to enter <0.05; *p* of F to remove >0.05); goodness of fitting was quantified using R^2^ adjusted, that quantify what % of the variability in the DV is accounted for by all of the IVs together, and the MSE (mean square error). Post-hoc power analysis was performed using *f*^2^ index. All the elaborations were performed using Microsoft Excel and SPSS 22.0 (IBM, Armonk, NY, USA).

## 3. Results

Means and range (min–max) of all variables are shown in Table 1.

The results of the multiple linear regression with backward stepwise elimination are presented in Table 2.

### 3.1. CMJ

Multiple linear regression was calculated to predict CMJ values. A significant regression equation was found (F_(5,16)_ = 9.86, *p* < 0.001), with an R^2^ adjusted of 0.679 (MSE = 3.43) considering, at the end of the backward stepwise procedure, only five variables (*f*^2^ = 2.11; large effect size). Age and IL17 were negatively associated with CMJ values, while IL-6, IL-10 and cortisol were positively associated. Participants’ predicted CMJ measurement was:

CMJ = 156.45 − 0.849 × Age + 0.548 × IL-6 − 0.427 × IL17 + 0.307 × IL-10 + 1.441 × Cortisol(3)

### 3.2. 10 m Sprint

A significant regression equation was found (F_(4,17)_ = 20.25, *p* < 0.001), with an R^2^ adjusted of 0.786 (MSE = 0.001) considering, at the end of the backward stepwise procedure, only four variables (*f*^2^ = 3.74; large effect size). Age and IL-10 were positively associated with 10 m sprint values, while TNF-α and haematocrit were negatively associated. Participants’ predicted 10 m sprint measurement was:

10 m sprint = 2.094 + 0.0136 × Age − 0.0016 × TNF + 0.0071 × IL-10 − 0.056 × Haematocrit(4)

### 3.3. VO_2_max

A significant regression equation was found (F_(9,12)_ = 32.09, *p* < 0.001), with an R^2^ adjusted of 0.930 (MSE = 0.558) considering, at the end of the backward stepwise procedure, nine variables (*f*^2^ = 13.0; large effect size). IL-6, erythrocyte, haematocrit, thrombocyte, PCR and testosterone were positively associated with VO_2_max values, while IL-10, leucocyte and cortisol were negatively associated. Participants’ predicted VO_2_max measurement was:

VO_2_max = −39.63 + 0.264 × IL-6 + 2.739 × Erythrocyte + 1.505 × Haematocrit + 0.048 × Thrombocyte + 76.55 × PCR + 41.224 × Testosterone − 0.161 × IL-10 − 1.145 × Leucocyte − 0.974 × Cortisol (5)

### 3.4. RSA Total Time

A significant regression equation was found (F_(4,17)_ = 15.31, *p* < 0.001), with an R^2^ adjusted of 0.732 (MSE = 0.667) considering, at the end of the backward stepwise procedure, only four variables (*f*^2^ = 2.73; large effect size). IL-6, erythrocyte and testosterone were positively associated with RSA values, while haematocrit and TNF-α were negatively associated. Participants’ predicted RSA measurement was:

RSA total time = 86.23 + 0.300 × IL-6 − 0.019 × TNF-α − 1.822 × Haematocrit + 19.14 × Testosterone(6)

### 3.5. RSA Index

A significant regression equation was found (F_(5,16)_ = 15.39, *p* < 0.001), with an R^2^ adjusted of 0.774 (MSE = 0.663) considering, at the end of the backward stepwise procedure, only five variables (*f*^2^ = 4.81; large effect size). Age, erythrocyte, thrombocyte, PCR and testosterone/cortisol ratio (T/C ratio) were negatively associated with RSA index. Participants’ predicted RSA index measurement was:

RSA index = 88.46 − 0.349 × Age − 2.648 × Erythrocyte − 0.004 × Thrombocyte − 46.22 × PCR − 78.84 × T/C ratio(7)

**Figure 1:**
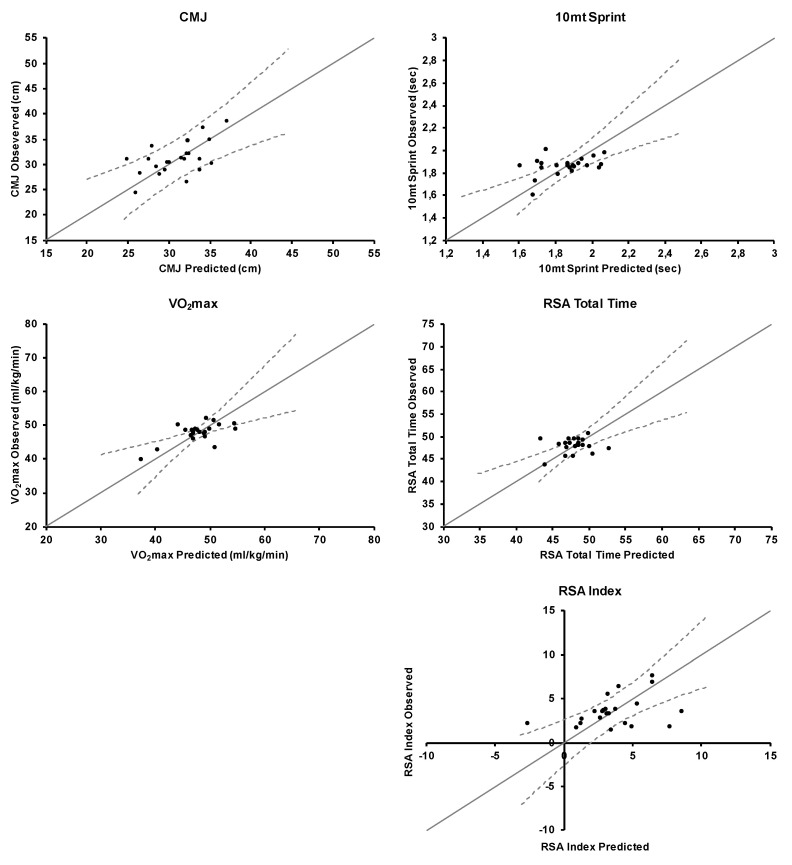
Correlations between predicted (*x*-axis) and observed (*y*-axis) values of fitness parameters. Dashed lines refer to 95% prediction bands.

Distributions of observed and predicted values for each dependent variable are presented in Figure 1.

## 4. Discussion

Training sessions and competitions induce physiological changes in soccer players, who require specific strategies to optimise efficiency, monitor overload and prevent the risk of injuries. The main finding of this observational study is that several physiological parameters can be predictors of fitness values in youth soccer players, at the end of the preseason soccer training period.

To examine specific predictive indices, previous reports [39,40,41,42,43] have assessed numerous variables (i.e., physical, anthropometric, technical and psychological) that may differentiate between selected vs non-selected athletes or successful vs unsuccessful athletes. Markers such as C, T, CK, sex hormones, cytokines, haematological panels and nutritional markers have been frequently used to assess athletes’ response to training load [20,44,45,46,47]. However, most previous attempts to predict the athlete’s response to a competitive season were probably limited by the interindividual variability, which requires repeated evaluations for each player during a prolonged period of physical stress [14,48,49]. The use of performance testing (CMJ, 10 m sprint, VO_2_max and RSA) and common biomarkers (blood-based molecules and salivary hormones), doubtless, might provide systematic monitoring across a competitive season.

Here we analysed youth soccer players before the championship, but after a strenuous pre-seasonal training period in order to match each profile with the overall trends measured in the whole team. For the first time of our knowledge, the single youth soccer player’s performances in the general context of the soccer team were evaluated. For this reason, our data were gathered only once during the preseason, after a brief but intense training load, potentially able to influence systemic and performance parameters, but presumably in a different way in every subject. On the other hand, the possibility of monitoring physical efficiency through the measurement of biochemical markers gave contradictory and cryptic results. For example, creatine kinase (CK) is considered a marker of skeletal muscle damage, which typically increases after a session of training [48,50]. Nevertheless, great interindividual variability in serum CK has been observed, which complicates its usefulness [5]. C and T, commonly measured in conjunction, are generally modified by acute as well as prolonged physical stress, but their possible correlation with an optimal fitness level is not automatic [5,12,47]. Haematocrit increase following a prolonged period of training is a matter of debate [5,51], whereas the exercise-induced muscle stress is a well-accepted mechanism of pro- (e.g., IL-6; TNF-α) and anti- (e.g., IL-10) inflammatory cytokine production [4,14,18,21,52]. For each performance test (CMJ, 10 m sprint, VO_2_max and RSA), we found a significant regression equation, suggesting a link between performance value and biochemical markers. In the light of these observations, our linear regression model suggests that the levels of such biomarkers might predict the results of physical performance tests, simplifying their interpretations.

CMJ values resulted positively associated with IL-6, IL-10 and cortisol, which means that their simultaneous increase should predict an improvement in the ability to perform such an explosive vertical jump. In the same way, better efficiency in 10 m sprint, VO_2_max and RSA resulted in correlation to other markers. Moreover, some biochemical parameters can be induced by physical stress (e.g., IL-6 and cortisol), whereas other markers (e.g., IL-10 and haematocrit) may be considered an adaptive response of our body in the presence of increasing training loads.

A further interpretation of our data involves the possibility to evaluate the player’s individual capacity to maintain physiological homeostasis, between anabolic and catabolic processes, throughout a whole tournament. In our opinion, the athletes with satisfactory results in the physical tests may have more possibilities to last over the competitive season, if biomarker perturbations do not occur. For example, optimal performances accompanied by growing levels of pro- (IL-6, TNF-α and IL-17), not counteracted by anti-inflammatory cytokines (IL-10), might induce overtraining status with a potential decline of fitness efficiency over time. Furthermore, significant alterations of the physiological homeostasis can trigger a vicious circle not only worsening the competitive results but also increasing the risks of possible injuries.

In a practical perspective, this is the first attempt done in the scientific literature to analyse youth soccer players by a multifactorial approach linked performance and biochemical parameters. Following the above rationale, results of our study suggest that the analysis of multifactorial indicators can be useful to monitor the levels of players’ physical performance by trainers and sports physicians, throughout the competitive season and in particular situations such as during recovery from injuries and in the off-season phase. Authors agree that the current paper can be used in the following three areas: (i) talent identification; (ii) performance and (iii) injury prevention.

Talent identification is composed of multidimensional performance factors including physical, physiological, technical, tactical, psychological and sociological influences [53,54,55,56,57], and it is important because selection and non-selection can have significant impacts on the careers and life directions of the athletes’ [58] and the success and direction of clubs. The coaching method of talent identification rests on intuitive knowledge comprised of socially constructed “images” of the perfect player. In addition to technical and tactical characteristics, our research can give information about the physical fitness of youth soccer players by simple haematological analyses.

It has been highlighted that soccer injuries affect: (1) performance [59,60], (2) economic aspects of a club [61] and (3) players’ health [62]. So, it is important that technical staff obtain objective information about the players’ physical performances to clarify the objectives of training, to determine whether an athlete is adapting to the training program and to minimise the risk of injury. Such information can be obtained by using tests that evaluate physical performance capacity. However, considering that during the competitive season it is difficult to propose a battery of a functional test (i.e., congested calendar and athlete injured) to predict a physical fitness value using a simple formula derived by a haematological analysis can be useful to the coaches to develop the training and/or recovery process adequate to each player.

Understandably, our study was subject to several limitations. First, the population was not very large. Second, we only analysed youth soccer players of the same category and chronological age. Third, results depend on the reproducibility of the variables analysed. For these reasons, further studies are recommended to ascertain the validity of the utilisation of a multifactorial approach in the youth of different ages, in adults and female soccer players. Implementation of additional monitoring tools, such as blood biomarkers, can give insight regarding athlete health, performance and recovery status by encompassing both on and off the field stressors.

## 5. Conclusions

Our observational study found significant correlations among haematological, inflammatory and endocrinological markers and fitness parameters. These results may contribute to assess the physical attitude of every player of a soccer team in each moment, and in each condition, of the agonistic season. We suggest the use of regression equations to estimate athletes’ attitudes in the general context of the team, in order to limit the risk of under-performances and possible injuries.

## Figures and Tables

**Table 1:** Means and range (Min–Max) and CV% of all variables analysed.

Variables		Mean ± SD	(Min–Max)	CV%
Dependent variables	CMJ (cm)	30.98 ± 3.27	(24.30–38.40)	10.5%
	10 m sprint (s)	1.86 ± 0.08	(1.60–2.00)	4.5%
	VO_2_max (mL/kg/min)	47.94 ± 2.83	(40.10–52.19)	5.9%
	RSA Total time (s)	48.06 ± 1.58	(43.70–50.70)	3.3%
	RSA Index	3.61 ± 1.71	(1.50–7.70)	47.5%
Independent variables	Age (months)	161 ± 3	(155–165)	2.0%
	BMI (kg/m^2^)	19.37 ± 2.16)	(16.15–23.63	11.1%
	IL-6 (pg/mL)	11.09 ± 5.40	(1.00–24.00)	48.7%
	IL-10 (pg/mL)	15.76 ± 6.91	(0–31.00)	43.8%
	IL-17A (MFI)	6.88 ± 5.18	(1.00–28.00)	75.2%
	TNF-a (pg/mL)	51.59 ± 65.30	(4.00–219.00)	126%
	Erythrocytes (10^6^/mL)	5.38 ± 0.53	(4.72–6.91)	9.8%
	Leucocytes (10^3^/mL)	6.15 ± 1.30	(3.54–9.26)	21.1%
	Thrombocytes (10^3^/mL)	235 ± 50.6	(134–378)	21.5%
	Haemoglobin (g/dL)	14.66 ± 0.78	(12.90–16.20)	5.3%
	Haematocrit (%)	43.58 ± 2.04	(39.80–49.30)	4.7%
	C-reactive protein (mg/dL)	0.03 ± 0.02	(0.01–0.08)	72.8%
	Lactate Dehydrogenase (U/L)	273 ± 50.7	(186–422)	21.5%
	Creatine kinase (mg/dL)	350 ± 258	(99–1081)	73.6%
	Cortisol (ng/mL)	2.40 ± 1.56	(0.84–7.64)	64.9%
	Testosterone (ng/mL)	0.06 ± 0.04	(0.01–0.20)	71.0%
	T/C Ratio (ng/mL)	0.03 ± 0.02	(0–0.9)	64.5%

**Table 2:** Multiple linear regression with backward stepwise elimination.

Dependent Variable	Independent Variables	Standardised 𝛽	Standard Error	t Value	*p* > |t|
CMJ	Age	−0.832	0.265	−3.146	0.006
	IL-6	0.906	0.399	2.271	0.037
	IL17	−0.677	0.269	−2.519	0.023
	IL-10	0.649	0.333	1.949	0.069
	Cortisol	0.687	0.288	2.384	0.030
10 m sprint	Age	0.523	0.216	2.419	0.027
	TNF-α	−1.271	0.399	−3.187	0.005
	Haematocrit	−1.379	0.606	−2.277	0.036
	IL-10	0.591	0.272	2.171	0.044
VO_2_max	IL-6	0.505	0.186	2.714	0.019
	IL-10	−0.393	0.155	−2.534	0.026
	Erythrocyte	0.512	0.249	2.056	0.062
	Haematocrit	1.084	0.346	3.135	0.009
	Thrombocyte	0.859	0.216	3.971	0.002
	PCR	0.629	0.126	4.987	<0.001
	Leucocyte	−0.526	0.169	−3.104	0.009
	Cortisol	−0.536	0.134	−3.993	0.002
	Testosterone	0.629	0.141	4.460	0.001
RSA Total Time	IL-6	1.030	0.365	2.823	0.012
	TNF-α	−0.798	0.447	−1.786	0.092
	Haematocrit	−1.236	0.678	−1.822	0.086
	Testosterone	0.524	0.276	1.895	0.075
RSA Index	Age	−0.652	0.222	−2.941	0.010
	Erythrocyte	−0.817	0.448	−1.822	0.087
	Thrombocyte	−1.301	0.389	−3.343	0.004
	PCR	−0.626	0.227	−2.761	0.014
	T/C ratio	−0.932	0.342	−2.721	0.015
